# Surgical management of female paraurethral cyst with concomitant stress urinary incontinence

**DOI:** 10.1590/S1677-5538.IBJU.2014.0582

**Published:** 2017

**Authors:** Tarik Yonguc, Ibrahim Halil Bozkurt, Salih Polat, Serkan Yarimoglu, Ismail Gulden, Volkan Sen, Suleyman Minareci

**Affiliations:** 1 Department of Urology, Izmir Bozyaka Training and Research Hospital, Izmir, Turkey

## Abstract

Paraurethral cysts are usually asymptomatic and frequently detected incidentally during routine pelvic examination, however, patients can present with complaints of a palpable cyst or with lower urinary tract symptoms (LUTS) and also dyspareunia. In most cases, diagnosis can be made on physical examination but for more detailed evaluation and to differentiate from malign lesions ultrasonography (US), voiding cystourethrogram (VCUG), computerized tomography (CT), or magnetic resonance imaging (MRI) can also be used. Management of symptomatic paraurethral cyst is surgical excision.

In this video our objective is to show the surgical management of female paraurethral cyst with concomitant stress urinary incontinence (SUI).

A 37 year-old woman presented with an 8-year history of progressive urinary symptoms, consisting of dysuria, urinary frequency, urgency urinary incontinence, SUI and dyspareunia. Physical examination in the lithotomy position revealed a cystic lesion located in the left anterolateral vaginal wall. Also cough stress test for SUI was positive. Her preoperative ICI-Q, UDI-6, IIQ-7 and SEAPI scores were 16, 8, 9 and 18 respectively. Vaginal US revealed a solitary 2 cm paraurethral cyst, localized in the distal urethra. Pelvic MRI also revealed a benign cystic lesion in the distal urethra. The patient underwent surgical excision of the cyst and anterior colporrhaphy for SUI. At third month visit the patient was very satisfied. The ICI-Q, UDI-6, IIQ-7 and SEAPI scores were 0.

Sometimes the LUTS concurring with the parauretral cyst can be dominant. Herein we want to show that extra surgical procedures can be necessary with paraurethral cyst excision for full patient satisfaction.

## ARTICLE INFO

**Available at:** http://www.intbrazjurol.com.br/video-section/20140582-yonguc_et_al Int Braz J Urol. 2017; 43 (Video #18): 1194-1194

